# Mast Cell-Mediated and Associated Disorders in Pregnancy: A Risky Game with an Uncertain Outcome?

**DOI:** 10.3389/fimmu.2014.00231

**Published:** 2014-05-19

**Authors:** Katja Woidacki, Ana Claudia Zenclussen, Frank Siebenhaar

**Affiliations:** ^1^Experimental Obstetrics and Gynecology, Medical Faculty, Otto-von-Guericke University, Magdeburg, Germany; ^2^Department of Dermatology and Allergy, Allergie-Centrum-Charité, Charité-Universitätsmedizin Berlin, Berlin, Germany

**Keywords:** mast cells, pregnancy, urticaria, PUPPP, mastocytosis, atopic dermatitis, asthma, psoriasis

## Abstract

During pregnancy, the maternal organism is under the influence of tremendous endocrine as well as immunological changes as an adaptation to the implanted and developing fetus. In most cases, the maternal adaptations to pregnancy ensure both, the protection against harmful pathogens and the tolerance toward the growing semi-allogeneic fetus. However, under certain circumstances the unique hormonal milieu during pregnancy is causative of a shift into an unfavorable direction. Of particular importance are cellular disorders previous to pregnancy that involve cell types known for their susceptibility to hormones. One interesting cell type is the mast cell (MC), one of the key figures in allergic disorders. While physiological numbers of MCs were shown to positively influence pregnancy outcome, at least in mouse models, uncontrolled augmentations in quantity, and/or activation can lead to pregnancy complications. Women that have the desire of getting pregnant and been diagnosed with MC mediated disorders such as urticaria and mastocytosis or chronic inflammatory diseases in which MCs are involved, including atopic dermatitis, asthma, or psoriasis, may benefit from specialized medical assistance to ensure a positive pregnancy outcome. In the present review, we address the course of pregnancy in women affected by MC mediated or associated disorders.

## Introduction

Pregnancy represents a unique challenge for the maternal organism. Tremendous endocrine and immunological modifications that occur as an adaptation to the implanted embryo ensure a successful pregnancy outcome. These necessary changes in the hormonal state and the shift toward anti-inflammation can, however, cause a dysregulations in the number and behavior of mast cells (MCs). MCs have been shown to exhibit beneficial function in pregnancy by contributing to implantation, placentation and fetal growth through their release of the glycan-binding protein galectin-1 and, thus, are critically implied in the fetomaternal interface (1). In addition, MCs influence pregnancy by modulating non-immunological responses by contributing to tissue remodeling, angiogenesis, and spiral artery modifications (1). In later pregnancy phases, however, MCs display rather detrimental functions as an excessive release of MC-mediators in parturition are associated with pre-term delivery. MC activation is modulated by hormonal endocrine signals that could lead to altered functional behavior of MCs in various innate and adaptive immune responses (1). In particular, pre-existing MC mediated and associated disorders may affect disease progression and the disease itself may influence pregnancy outcome. Sex hormones are considered to influence the clinical course and severity of chronic allergic and inflammatory diseases, including atopic dermatitis (AD), asthma, and psoriasis (2–5). In fact, estrogen and progesterone have been reported to modulate tissue homeostasis and immunological responses in various conditions.

Here, we review the existent literature on the clinical implications of MC associated disorders in pregnancy, focusing on directly MC mediated disease such as urticaria and mastocytosis, but also summarize the disease impact on pregnancy of common inflammatory disorders in which MC have been reported to critically contribute to the pathogenesis. Hereby, we offer an overview of how disease-specific modifications in MC function and activation could determine the fate of pregnancy.

## Role of Mast Cells and Mast Cell Mediators in Pregnancy

Mast cells reside in the endometrial tissue and uterine MCs exhibit signs of activation during premenstrual stages (6). MCs granules consist of a large array of mediators, including histamine, prostaglandins, leukotrienes, several cytokines, and proteases (7). The release of MC protease such as tryptase has been reported to stimulate the production of matrix metalloproteinases (MMPs) that are involved in the degradation of extracellular matrix components. Increase protease expression is detected during menstruation (8). Histamine, which is produced and released by MCs, has been reported to be involved in blastocyst implantation and placenta development by contributing to and promoting trophoblast invasion, growth, and the expression of adhesion (9, 10) molecules. During pregnancy, the number of MCs increase in the myometrium and equal ratio of tryptase and chymase (MC_TC_) positive MCs shift toward a tryptase-only (MC_T_) phenotype (11). Here, histamine, prostaglandins and MC proteases have been shown to contribute and modulate myometrical contractility (12, 13). MC proteases may also be involved in post-partum uterine tissue remodeling (14). Given these facts, it is reasonable to suggest that MCs influence pregnancy outcome under physiological conditions and even more relevant when their activation status is modulated by disease. The prevalence of MC mediated and associated disorders, including allergic and non-allergic diseases, is increasing. Severe allergic reaction, i.e., anaphylaxis during pregnancy can indeed result in pre-term labor whereas adequate treatment, including antihistamines and corticosteroids, reportedly inhibited uterine contractions (15). This evidence points toward the importance of disease management of MC-related disorders during pregnancy. Although, the use of systemic treatments should be limited or even generally avoided in pregnancy – especially in the first trimester, pregnant women require best possible treatment. But how to provide best possible treatment by calculated risk profile? The use of second-generation antihistamines (sgAH) for example is widely used for the treatment of allergic disease. In pregnancy, it is recommended to limit the use of sgAH to loratadine (16), and possibly desloratadine, because of best available evidence. Nowadays, several AH are OTC (over-the-counter) products in many countries and it can be assumed that these drugs are frequently used by pregnant women – at least before they knew to be pregnant. However, up-dosing of sgAH as it is recommended in the management of chronic urticaria must be carefully suggested in pregnancy since safety studies have not been performed (17).

## Urticaria in Pregnancy

Urticaria is a very common dermatological condition in which an increased activation of MCs and the subsequent release of MC-mediators, mainly histamine among others, lead to the development of wheal-and-flare responses accompanied by an intense pruritus on the skin (18). Urticaria is divided into acute (less than 6 weeks) and chronic (more than 6 weeks) forms as well as into inducible and spontaneous occurring subtypes (17). Urticaria may develop during pregnancy even though it is not considered as a specific pregnancy dermatosis. MCs have also been reported to contribute to the pathogenesis of pregnancy-related dermatoses associated with pruritus that are restricted to pregnancy, e.g., pruritic urticarial papules and plaques of pregnancy (PUPPP). Urticaria can either develop during pregnancy or the symptoms of a pre-existing chronic spontaneous urticaria (CSU) may change in terms of disease activity and severity. Influence of sex hormones on MC functions and the pathogenesis of CSU have long been considered (19). CSU is approximately twice more frequent in women than in men and the disease activity of CSU may be associated or triggered by fluctuation of sex hormone levels, including menstrual cycle, pregnancy, menopause, and hormonal therapies (19). CSU may worsen with pregnancy in some patients but also improve in others (20). Hypersensitivity to sex hormones and their modulating actions on MCs have been implicated in the pathogenesis of CSU and altered hormone serum levels have been described in subgroups of CSU patients (21). Thus, such fluctuations in the hormonal milieu have been suspected to either improve, maintain, or aggravate urticarial lesions during pregnancy (19). PUPPP and other pregnancy-related dermatoses associated with pruritus should be considered as differential diagnosis if wheals and itch newly occur in pregnancy, especially during the third trimester (22). In addition, special treatment considerations should be applied to pregnant and lactating women (17, 23).

## Pruritic Urticarial Papules and Plaques of Pregnancy

Pruritic Urticarial Papules and Plaques of Pregnancy or polymorphic eruption of pregnancy (PEP) is the most common pregnancy-related skin disorder with an incidence of about 1:160–1:200 (24). This disease appears and exists exclusively in pregnant patients. Besides the pruritic urticarial papules and plaques, which represent the key symptoms of PUPPP, more than one-half of the patients later develop polymorphous features including erythema, vesicles as well as targetoid and eczematous lesions (25). Characteristically, the eruptions begin on the abdomen, particularly, within or adjacent to *striae cutis distensae* and occur predominantly in the third trimester in about 83% of the patients (25–27). It is suggested that multiple gestations and an excessive maternal weight gain is associated with the occurrence of PUPPP (25, 28). The fetal weight and sex does not seem to be related to the onset of PUPPP (25). Cortisol serum levels have been found to be significantly reduced in PUPPP patients whereas estradiol concentrations were comparable with unaffected women (27). It is tempting to speculate that MCs are involved in the onset of PUPPP although no studies are existing showing a direct link between MCs and this disease. It is suggested that the activation of the skin immune system characterized by increased numbers of dentritic cells and activated T cells in lesional skin contribute to the pathology of PUPPP (29). Skin infiltrates of macrophages (30) and eosinophils (25, 27) have been described in affected tissue. Although, a direct implication of MCs has, as of yet, not been reported in PUPPP there are several lines of evidence that clearly suggest a role for MCs. First, as in urticaria, antihistamines are the first line option in the treatment of PUPPP and are effective in most patients. MCs are considered as the main source of histamine in the skin (31). Second, even though PUPPP and urticaria are different diseases there are several similarities in terms of the clinical symptoms including pruritic erythema and urticarial lesions. Third, autologous whole blood injections have been reported as an effective treatment option in PUPPP as it is in auto-reactive CSU (32, 33). Therefore, even if still speculative it is not unlikely that the release of MC-mediators critically contribute to the pathogenesis of PUPPP.

## Mastocytosis and Pregnancy

Mastocytosis represents a group of related disorders, each characterized by a pathological accumulation of MCs in one or more organs ranging from indolent to very rare aggressive forms (34). Mastocytosis is classified as a rare disease with an estimated prevalence of around 1 per 10,000 and dividing cutaneous from systemic forms (35). About 80% of mastocytosis patients carry an Asp816Val mutation in the catalytic domain of the c-Kit receptor downstream tyrosine kinase in peripheral blood mononuclear cells (36). This point mutation mediates an increased proliferative rate of MCs (37). An addition explanation for the increased numbers of MCs in tissues from mastocytosis patients might be the enhanced chemotaxis of CD117 positive cells. It is speculated that MC progenitor cells bearing the D816V mutation preferentially migrate to SCF produced by stroma cells, endothelial cells, fibroblasts, and keratinocytes in the skin (38). It could be shown that MCs present in the lesions express SCF suggesting a potential autocrine or paracrine growth and differentiation loop for MCs and lymphoid progenitors (39, 40). Differentiation of the mutant progenitor cells into mature MCs occurs locally based on the specific microenvironment. Thus, enhanced MC migration combined with aberrant proliferation contribute to the extensive MC hyperplasia observed in affected tissues (38). Beside the elevated serum tryptase levels in mastocytosis patients (41), the coexpression of CD25 antigen in bone marrow MCs turned out as diagnostic marker in mastocytosis (42, 43). In bone marrow biopsies from mastocytosis patients, MCs are surrounded by lymphoid aggregates, which consist of a mixture of B and T cells (39, 44). Similar to MCs, these B and T cells carry the D816V mutation (44).

Between 20% and one third of pregnant women with mastocytosis reported a worsening of the diseased-related symptoms (45, 46). Around 30% experienced a clinical improvement during the first trimester. In the other half of the affected population, MC-mediator related symptoms remained unchanged (46). Interestingly, worsening of symptoms was observed during the first or third trimester (46) when Th1-mediated pro-inflammatory conditions dominate (47–49). Although, women diagnosed with mastocytosis are often required to continue the intake of medications including antihistamines during pregnancy the doses are often decreased because of fetal safety concerns (45). The reduction in medication as well as an irregular medication intake could contribute to worsening of mastocytosis symptoms as well. Undiagnosed and not appropriately treated mastocytosis can be associated with severe pregnancy complications including fetal demise (50).

Parturients suffering from mastocytosis that do not undergo a natural birth represent a particular challenge for anesthesiologists. During the process of labor, life-threatening complications may occur, particularly due to the risk of anaphylactoid reactions triggered by anesthesia. Medications such as glucocorticoids, antihistamines, and epinephrine should be available during the critical phases of labor and the early post-partum period (51).

In general, studies describing the impact of mastocytosis in pregnancy and vice versa are limited. Thus, one can only speculate that the unique pregnancy-associated micromilieu composed of hormones and myriads of mediators contribute to variations in the disease pattern.

## Atopic Dermatitis in Pregnancy

Atopic dermatitis is a complex chronic inflammatory condition in which MCs have been shown to contribute critically to the pathogenesis and the induction of inflammation and pruritus. MC number and degranulation is increased in atopic lesions (52). It is suggested that the invasion and degranulation of MCs within peripheral nerve bundles may provoke and aggravate itchiness of AD (53). MC-derived mediators might participate in epidermal hyperplasia seen in lichenified lesions in AD (54).

**Figure 1:**
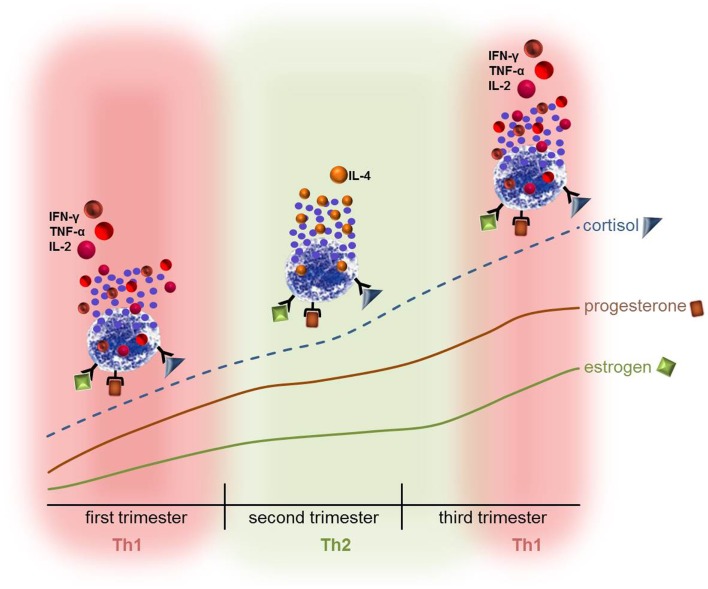
**The hormonal stimulation of mast cells during pregnancy might contribute to the Th1/Th2 switch that takes place in pregnancy**. The ever-going increase in estrogen, progesterone, and cortisol could directly influence the activation status and behavior of MCs and lead to the release of either pro- or anti-inflammatory mediators thus contributing to the Th1 or Th2-based micromilieu. Hence, disorders that are mediated by MCs or in which MCs are involved may turn into an unfavorable direction. It is however also possible that the symptoms ameliorate due to the hormone-modulated behavior of MCs.

Atopic dermatitis is one of the most prevalent dermatoses during pregnancy (55, 56) and pregnancy may alter the clinical course and severity of AD. More than half of pregnant women with pre-existing AD were reported to experience worsening of their disease during the second or third trimester (57, 58) when a constant Th2 response is maintained. During pregnancy an immunologic homeostasis tolerating the fetus is of crucial importance. To prevent fetal rejection, maternal T cell mediated immunity is modulated. AD is widely accepted as a Th2-dominated disorder. Therefore, alteration in the Th1/Th2 balance is suggested to promote AD severity, which is often observed in pregnancy (59). MCs found in AD lesions are a major source of IL-4 and store higher amounts of IL-4 compared to MCs in normal skin (60). Exogenous IL-4 has been shown to be important for the differentiation of T helper cells into Th2 cells (61). Thus, one can speculate that MCs participate in the Th1/Th2 switch and therefore disease severity (Figure 1). Moreover, IL-4 induces the proliferation of fibroblasts (62) and atopic fibroblasts contribute to the pathogenesis of AD by initiating strong proliferation and differentiation defects in keratinocytes (63).

Variations in sex hormone concentrations seem to be related with the severity of AD that is supported by the finding that ca. one third of women reported a premenstrual deterioration in the symptoms (57, 58). Sex hormones may also directly influence AD symptoms by their effects on MCs, mediator release, and IgE production (64) (Figure 1). No reported evidence point toward direct influence of AD on infertility or increased rates of miscarriage, birth defects, or prematurity (65). However, AD patients that are more prone to suffer from bacterial or viral super-infection may be at a higher risk for birth complications including premature delivery, intrauterine growth restriction, or miscarriage (66). The application of large doses of triamcinolone acetonide for treatment of AD should be avoided as it was related with intra uterine growth retardations (67). Therefore, specific considerations about the treatment strategies in AD during pregnancy will apply to control the impact of AD progression and complications that may occur on pregnancy.

## Asthma and Pregnancy

In 2004, the number of people affected by asthma worldwide was estimated as 300 million with a further increase up to 400 million asthmatics by 2025. The rate of asthma increases as communities adopt western lifestyles and become urbanized (68). It is assumed that environmental estrogens participate in the development of asthma as they induce MC degranulation via the estrogen receptor-α. These pollutants show estrogen-like activities, tend to degrade slowly, have a long biological half-life, and bioaccumulate and bioconcentrate in the food chain (69).

Asthma is characterized by recurrent episodes of airway obstruction, which reverse either spontaneously or after use of medication. It is usually associated with bronchial hyper-responsiveness and evidence of chronic airway inflammation (70). The earliest text where the term “asthma” was mentioned in a medical context is in the Corpus Hippocraticum. Several centuries later during the second half of the first century A.D. Aretaeus the Cappadocian was the first one who dealt with asthma as an autonomous clinical disease and not as a symptom (71). One characteristic of asthma is the exaggerated narrowing of the airways that is caused by contraction and shortening of airway smooth muscle (ASM) cells. However, the cause of the induced displacement behavior of the ASM is still a matter of discussion. It is proposed that following processes could contribute to the SMC response: (1) changes in ASM structure and/or behavior; (2) structural and/or mechanical alterations in the non-contractile structures of the airway wall, and (3) variations in the relationship of the airway wall to the surrounding lung parenchyma (72). The airway remodeling observed during the course of asthma includes the increase in the smooth muscle cells surrounding the airway wall, a deposition of extracellular matrix components under the epithelial basement membrane that causes a thickened appearance, a breach in the integrity of the airway epithelium and an increase of mucus-producing goblet cells in the epithelium or submucosal glands (73).

Various immune cell types including macrophages, eosinophils, and MCs participate in the process of airway remodeling; accordingly they were found in high numbers in bronchoalveolar lavage and in bronchial biopsies from asthmatic patients (74). Asthma is i.a. characterized by an infiltration of MCs in the bronchial epithelium (75), mucous glands, and smooth muscle (76, 77). It is hypothesized that the ASM itself induce the migration of MCs and their progenitors via the release of the chemoattractants CXCL9, CXCL10, CXCL11, stem cell factor (SCF), and transforming growth factor (TGF)-β (78–80). ASM would also induce MC proliferation and survival (81). Once resident in the ASM bundle, MCs adhere to it via cell adhesion molecule 1 (CADM1) (82). The release of MC-mediators such as histamine, prostaglandin D_2_, and leukotriene C_4_ upon activation induces typical asthmatic symptoms including bronchoconstriction, mucus secretion, and mucosal edema (80). It is often assumed that the activation of MC during asthma is allergen-dependent via the high affinity IgE receptor FcεRIα. Mucosal MCs present in bronchial tissue from asthmatic patients exhibit features of chronic activation (83).

About 1% of pregnant women are diagnosed with active asthma (84). The inflammatory response induced in asthmatic airways contributes to the pathophysiology of this disease (85). This in turn could interfere with the necessary variations in the cytokine milieu mandatory for pregnancy to occur and be maintained. The first trimester and also the early phases of the second trimester of pregnancy require a strong inflammatory response in order to ensure uterine tissue remodeling and clearance of cellular debris (47–49). During the course of pregnancy, the concentrations of the steroid hormones estradiol (E_2_) and progesterone (P_4_) raise more than fivefold followed by a further fivefold increase by term (86). The immunological shift toward Th1 responses at the beginning of pregnancy is mediated by enhancing pro-inflammatory cytokine production by monocytes and macrophages (87), dendritic cells (88) as well as MCs (89) (Figure 1). The ever-going increase in estrogen, progesterone, and cortisol concentrations at midpregnancy supports a shift toward Th2 responses, which are necessary for maintaining pregnancy (90). As asthma is a classical Th2-driven disease its course may be negatively influenced by the establishment of an anti-inflammatory milieu as it is observed in the second and early third trimester (91). Indeed, during pregnancy asthma worsens in 35% of the women with an increase in asthma symptoms starting at the third trimester (92) when a constant Th2 milieu was established. MCs express the receptors for E_2_ and P_4_ (93, 94) and degranulate upon treatment with theses hormones (94). Based on these findings, one might speculate that the rising levels of estrogens and progesterone during pregnancy stimulate MCs to degranulate (Figure 1) whereby they could negatively influence the course of asthma. This could be an explanation of why one third of women present a worsening of asthma symptoms at midpregnancy (92). However, 28% of the women experienced an improvement of asthma symptoms and further 33% showed no changes (92). Women who reported changes in their asthmatic symptoms during pregnancy reverted post-partum toward their pre-pregnancy asthma course (92).

An elegant study by Perlow et al. revealed that pregnant women requiring long term administration of oral steroids are at an increased risk of pre-term labor and delivery as well as to develop gestational diabetes (95). Moreover, steroid-dependent (95, 96) but also non-steroid medicated (95) asthmatic mothers delivered more often low birth weight neonates with less than 2500 g than mothers without asthma. Intriguingly, the boost in asthma symptoms during pregnancy seems to correlate with the gender of the fetus. While women who delivered boys reported an improvement in their asthma during their pregnancy, mothers of girls, however, had increased asthma severity during gestation (97, 98). Women who were pregnant with a female fetus needed significantly more inhaled glucocorticoids in late pregnancy. For this particular group, it is proposed that an upregulation of inflammation is associated with asthma as gestation progressed (98). The mechanism behind is not entirely understood. In asthmatic mothers pregnant with female fetuses, the placental activity of the enzyme 11-hydroxysteroid dehydrogenase type 2 (11-HSD2) that metabolizes cortisol to inactive cortisone is reduced (98). This would increase the intracellular cortisol concentration in relation to variations in cytokine production in female placentas compared to male placenta explants (99). It was reported that the inflammatory response of placental trophoblast cells from male pregnancies after LPS stimulation is boosted probably because of the enhanced toll-like receptor (TLR)-4 expression in these cells (100). These fetal gender-specific differences should be taken into account during pregnancy in asthmatic women.

## Psoriasis and Pregnancy

About 25 million people in Europe and North America are affected by psoriasis that counts to the most prevalent immune-mediated skin disease in adults (101). Psoriasis is considered to be a genetically programed, organ-specific (skin, or skin and joints) inflammatory disease (102) characterized by red, scaly, and raised plaques (101). Vascular dilation, bridged fenestrations, and gaps in endothelium, edematous areas in the cytoplasm of endotheliocytes, myocytes, and pericytes, basement membrane zone thickening and cell extravasation are reported as microvascular changes that occur in psoriatic lesions and represent signs of increased vascular permeability (103). Compared to asthma, psoriasis is considered to be a Th1-driven disease characterized by the infiltration of several T cell subsets, neutrophils, dendritic cells, natural killer T cells, and MCs (102, 104). All of them contribute to the inflammatory microenvironment that is composed of increased levels of interferon (IFN)-γ, tumor necrosis factor (TNF)-α, IL-2, and IL-12 (105) as well as IL-23 and IL-17A. MCs have been demonstrated to be key modulators of T-cell mediated responses and essentially involved in neutrophil recruitment (106). Recently, the important role of IL-17 producing Th17 cells in the pathogenesis of psoriasis has been reported (107). Neutrophils and MCs are other significant potential sources of IL-17A in psoriasis (108). In the superficial dermis of psoriatic skin, MC density is increased (104). It has been shown that alterations in psoriatic tissue appear to be initiated by degranulating MCs (109) and MC degranulation is among the earliest events in relapsing psoriasis lesions (109). Degranulated MCs have been found in close proximity to blood vessels in the area of psoriatic lesions (103). One can speculate that the survival of MCs in the tissue is regulated by their most important growth factor, the SCF that is intensely expressed in psoriatic tissue while its receptor KIT is upregulated in the surface of MCs (110).

Mast cells are typically classified into either MC_T_ that contain only tryptase or MC_TC_ that contain both proteases tryptase and chymase. Both subtypes in human are suggested to be equivalent to the murine MC phenotypes. While MC_T_ seem to be related to immunological processes, MC_TC_ appear to be linked to non-immunological responses including tissue remodeling and angiogenesis. The tryptase is the quantitatively dominant protease present in all MC phenotypes (111). In psoriatic skin, tryptase-positive cells are increased in number (112, 113). However, the determination of serum tryptase levels is not an appropriate tool to assess the severity of psoriasis as no correlation could be found between serum tryptase and psoriasis severity in patients (114). Tryptase levels in normal subjects are undetectable (<1 ng/ml) whereas in systemic MC disorders such as mastocytosis and anaphylaxis elevated tryptase levels can be detected (41).

It was reported that approximately 50% of patients develop psoriasis before the age of 25 (115). There are several studies showing that the natural course of psoriasis in women is modulated by menstrual cycle, pregnancy, and menopause (5, 115, 116). All of these events during the reproductive cycle are under hormonal regulation. During pregnancy, more than 50% of the women reported an improvement of psoriasis at ca. the 30th week of pregnancy (midpregnancy) while more than 20% observed a worsening (116). At this time point, the shift from Th2 to Th1 immunity occurs mainly mediated by increased levels of estrogen, progesterone, and cortisol (90). It is assumed that during pregnancy and when hormone levels are increased, psoriatic symptoms improve. During puberty, post-partum and menopause when hormone levels decrease the disease severity seems to peak (117). Several Th1-characterized diseases including psoriasis (116), multiple sclerosis (118), and rheumatoid arthritis (119, 120) have been shown to improve during pregnancy. Psoriatic body surface areas (BSA) decreased significantly from 10 to 20 weeks’ gestation. Thereby, increased levels of estrogen relative to progesterone correlate with the improvement of psoriasis while progesterone concentrations alone did not correlate with changes in psoriatic symptoms. Interestingly, post-partum more than 60% of the patients reported worsening of symptoms while only 8.7% observed an improvement. But the authors found out that the “post-partum flare” was a return to the patients’ baseline, rather than a real worsening (116).

It was reported that pregnant psoriatic women are at increased risk to adverse pregnancy outcomes including spontaneous and recurrent abortion, gestational hypertension, ectopic pregnancy, and pre-term rupture of membranes (121, 122). In fact, study results are controversial. Some showed an increased risk of adverse pregnancy outcomes, others did not. Psoriasis is definitely no contraindication for a pregnancy but a well-controlled disease and the monitoring of comorbidities, such metabolic syndrome, during pregnancy is of advantage. In contrast, generalized pustular psoriasis of pregnancy (GPPP), a special subtype of psoriasis occurring during pregnancy might be harmful for mother and child (123). In such cases, women should be kept on medical care by dermatologists. Several medications are available whose suitability and unsuitability for the treatment of psoriasis during pregnancy are reviewed by Lam et al. (124). However, safety data are limited because most of the data are based on case reports, which lack the comparison group of untreated patients (124). Women who need a disease-specific treatment should keep in mind that leaving a disease untreated during pregnancy may carry a greater risk to both the mother and fetus than any teratogenic risk of the drug to the fetus (124). Systemic treatment should be avoided when possible, but if necessary the best treatment option should be chosen on a case to case basis for optimized treatment during pregnancy.

## Resume

In general, there is no contraindication to pregnancy when MC-related pathologies are under appropriate medical control. Women who were diagnosed with MC mediated or associated disorders and especially those whose disease is active, should be carefully advised by medical specialists to avoid severe pregnancy complications and to monitor disease progression. The unique modifications of the maternal endocrine and immune system can influence the number and behavior of MCs. Further studies addressing the molecular mechanisms behind the impact of pregnancy on MC mediated and associated disorders are needed in order to optimize pregnancy course and outcome.

## Conflict of Interest Statement

The authors declare that the research was conducted in the absence of any commercial or financial relationships that could be construed as a potential conflict of interest.
